# A UHPLC-MS/MS Method for the Detection of Meat Substitution by Nine Legume Species in Emulsion-Type Sausages

**DOI:** 10.3390/foods10050947

**Published:** 2021-04-26

**Authors:** Johannes Spörl, Karl Speer, Wolfgang Jira

**Affiliations:** 1 Department of Safety and Quality of Meat, Max Rubner-Institut (MRI), E.-C.-Baumann-Straße 20, 95326 Kulmbach, Germany; johannes.spoerl@mri.bund.de; 2 Faculty of Chemistry and Food Chemistry, Technical University of Dresden, Helmholtzstraße 10, 01069 Dresden, Germany; karl.speer@chemie.tu-dresden.de

**Keywords:** foreign protein, meat substitution, food adulteration, allergens, legumes, mass spectrometry, food fraud, food safety, marker peptides

## Abstract

Meat substitution by legume proteins in various types of meat products is a common practice. A reliable detection and quantification of these additives is required to control food specifications, especially regarding food fraud. Consequently, a UHPLC-MS/MS method for the simultaneous detection of alfalfa (*Medicago sativa*), broad bean (*Vicia faba*), chickpea (*Cicer arietinum*), lentil (*Lens culinaris*), lupine (*Lupinus albus* and *Lupinus angustifolius*), pea (*Pisum sativum*), peanut (*Arachis hypogaea*), and soy (*Glycine max*) proteins in meat products was developed. After protein extraction and tryptic digestion, three marker peptides for each legume species were measured by multiple reaction monitoring (MRM) using an optimized extraction protocol. To the best of our knowledge, the marker peptides for alfalfa, broad bean, chickpea, and lentil have not been reported previously. Emulsion-type sausages with 0.1, 0.4, 0.7, 1.0, 1.3, 1.6, 1.9, 2.2, and 2.5% meat substitution by each legume species, representing the concentration range between inadvertently transferred cross-contaminations and the conscious use for meat substitution, were produced for matrix calibration. No false-positive results were recorded in blank samples. In the quantification of alfalfa, broad bean, chickpea, lentil, pea, peanut, and soy, 673 of 756 measuring data of the recovery rate in unknown sausages were in the accepted range of 80–120%.

## 1. Introduction

The addition of foreign protein to a wide range of meat products such as emulsion-type sausages is a very common practice [1]. According to the German guiding principles for meat and meat products, foreign protein is defined as protein which is not derived from slaughtered or hunted warm-blooded animals [2]. The addition of foreign proteins to meat products must be stated in the list of ingredients according to Regulation (EU) No 1169/2011 [3]. The most frequently used foreign protein sources are soy protein, milk protein, and wheat gluten [1]. However, a variety of other sources exist, especially high-protein legumes, showing crude protein contents (related to the dry matter of ripe seeds, each) as follows: Soy (41%), lupine blue (40%), lupine white (40–45%), peanut (31%), lentil (29%), broad bean (27%), pea (26%), chickpea (23%), and alfalfa (18%) [4,5]. It should also be mentioned that the remaining residues of the production of peanut and soy oil (defatted materials) show significantly higher protein contents than the ripe seeds (peanut (55%) [6] and soy (49%) [7]). In addition to the high protein content of legumes, their high global production rates also favor their use as cost-effective sources of foreign protein in meat products. The production rates in 2018 were as follows: Soy (349 million tons), peanut (46 million tons), chickpea (17 million tons), pea (14 million tons), lentil (6 million tons), broad bean (5 million tons), and lupine (1 million tons) [8]. Even if no data are available for alfalfa, this legume species has been recognized as an alternative protein source for human consumption [9].

Within these legume species, soy, pea, and lupine are especially available as protein isolate or flour and are already used in the production of various foods, such as meat analogue products [10,11]. The addition of legume proteins to meat products is carried out due to economic and technological reasons such as the increase of the product’s water-binding capacity resulting in less water exudation during sterilization [12], for the improvement of textural properties [13,14], and to exploit the use of low-quality meat [1]. In addition, pea protein is added to meat products to manufacture hybrid meat products combining meat and vegetable protein [12,15].

Analytical methods are required for the detection of legume proteins in meat products. In this context, a fraudulent substitution of meat protein by legume proteins must be differentiated from an unintentional contamination with traces (e.g., via cross-contamination during production, contamination of spice mixtures, other food additives, or processing aids). However, the detection of traces is only relevant when considering the allergenic potential of the legumes lupine, peanut, and soy, which belong to the 14 main allergens of the Commission Directive 2007/68/EG [16].

Analytical methods for the detection of legumes in food in the scientific literature are restricted to lupine, peanut, and soy, belonging to the group of the 14 EU main allergens, and to a much lesser extent to pea. These methods are most commonly based on enzyme-linked immunosorbent assays (ELISA) [17,18], polymerase chain reaction (PCR) [17,18] or high-performance liquid chromatography—tandem mass spectrometry (HPLC-MS/MS) [19,20]. Commercial ELISA and nucleic acid-based kits are available for lupine, peanut, and soy [18]. In contrast to ELISA and PCR, mass spectrometric methods have the advantage of being high-throughput screening tools for the multiplex detection of target proteins in foods [19,21] and are less vulnerable to interferences in complex food matrices [19]. Only a few analytical methods exist for the HPLC-MS/MS-detection of legumes in meat products, which are limited to soy [22,23,24] or lupine, pea, and soy [25]. However, other legume species are also potential foreign protein sources in meat products, as mentioned before, for which no detection methods to uncover this type of meat adulteration have been available until now.

The main objective of this study was to develop a novel analytical UHPLC-MS/MS screening method for the simultaneous detection of proteins of the nine main legume species: Alfalfa (*Medicago sativa*), broad bean (*Vicia faba*), chickpea (*Cicer arietinum*), lentil (*Lens culinaris*), lupine (*Lupinus albus* and *Lupinus angustifolius*), pea (*Pisum sativum*), peanut (*Arachis hypogaea*), and soy (*Glycine max*) in meat products using characteristic tryptic marker peptides. In this context, on the one hand, the suitability of marker peptides for the legume species belonging to the 14 EU main allergens known from the scientific literature was checked. On the other hand, new marker peptides for the other legume species mentioned were identified. The focus of this method was the detection of the undeclared addition of legume proteins allowing a quantitation of the meat protein substitution by legume proteins in the range of 0.1–2.5%, which was performed applying a matrix calibration. Accordingly, the method should reliably detect the selected legumes in different concentration ranges in emulsion-type sausages and allow for a subsequent quantitative determination.

## 2. Materials and Methods

### 2.1. Materials

#### 2.1.1. Chemical Material

The solvents acetone, acetonitrile (ACN), and LC-MS/MS water were purchased from LGC Standards (Wesel, Germany) in Optigrade quality. Ethanol (absolute, p.A.), 2-propanol, hydrochloric acid (HCl), and formic acid (p.A.) were obtained from Merck (Darmstadt, Germany). Tris(hydroxymethyl)-aminomethane (TRIS; ≥99.3%) was purchased from Carl Roth (Karlsruhe, Germany), trypsin (sequencing grade) from Promega (Madison, WI, USA), and dimethyl sulfoxide (p.A.) from J.T. Baker (Center Valley, PA, USA). Formic acid (for LC-MS) was bought from Honeywell (Charlotte, NC, USA). The 9-fluorenylmethoxycarbonyl (Fmoc)-l-amino acids, the amino acid-wang-resins (Fmoc-l-Arg (Pbf)-Wang Resin, Fmoc-l-Lys (Boc)-Wang Resin), dimethylformamide, trifluoroacetic acid (Peptide grade), triisopropylsilane, diisopropylcarbodiimide, and piperidine for the peptide synthesis were obtained from Iris Biotech GmbH (Marktredwitz, Germany). 6-chloro-1-hydroxybenzotriazole (6-Cl-HOBt) was purchased from Luxembourg BioTechnologies (Ness Ziona, Israel), and methanol (for LC-MS) from Merck (Darmstadt, Germany). Iodoacetamide (IAA) and DL-Dithiothreitol (DTT; ≥98%) were bought from Sigma (St. Louis, MO, USA).

#### 2.1.2. Sample Material

Peanut flour (*Arachis hypogaea*), soy protein isolate (*Glycine max*), and pea protein isolate (*Pisum sativum*) were obtained from Bulkpowders (Colchester, UK). White lupine meal (*Lupinus albus*) was purchased from Govinda Natur GmbH (Neustadt an der Weinstraße, Germany). The flours of blue lupine (*Lupinus angustifolius*), chickpea (*Cicer arietinum*), lentil (*Lens culinaris*), and the seeds of alfalfa (*Medicago sativa*) were obtained from Rapunzel Naturkost GmbH (Legau, Germany). The seeds of broad bean (*Vicia faba*) were purchased from Bioland Hof Jeebel (Salzwedel, Germany). The pure seeds of lupine blue and lupine white were obtained from the Leibniz Institute of Plant Genetics and Crop Plant Research (IPK, Gatersleben, Germany). The pork meat and fat for the emulsion-type sausages were obtained from Emil Färber GmbH & Co. KG (Kulmbach, Germany). The nitrite pickling salt was purchased from Süddeutsche Salzwerke AG (Heilbronn, Germany) and phosphate powder (E450) was purchased from KKS Karl Konrad GmbH & Co. KG (Kirchheimbolanden, Germany).

The nine commercial legume materials mentioned above were homogenized and blended for the production of a flour mixture: (a) To obtain comparable concentrations of legume protein from each legume species for the sausage of processing series 1 (test sausages); and (b) to obtain a meat substitution for the sausages of processing series 2 (standard and unknown sausages), as shown in Table 1. Therefore, the protein concentration of each type of legume flour was determined in accordance with the respective method (protein determination according to Kjeldahl) [26] in the German Food and Feed Code (§64 LFGB) [27].

Different concentrations of the nine legume species were used consistently for the production of the emulsion-type sausages as shown in Table 1. The meat was minced in a meat grinder (Moulinex, Alencon, France). The sausages were produced in a 13 L bowl chopper (Müller Food Machines, Saarbrücken, Germany) and the maximum temperature of the meat was 12 °C. The sausage meat was stuffed into 200 g tin-plate cans (type 99/36 mm; Dosen–Zentrale Züchner GmbH, Wiesbaden, Germany). The sausages were heated (fully preserved, F-value = 5) and, subsequently, cooled overnight and stored at 2 °C.

### 2.2. Methods

#### 2.2.1. Sample Preparations for Mass Spectrometry

The sample preparations of (a) legume flours for high-resolution and (b) homogenized emulsion-type sausages for triple-quadrupole mass spectrometry were performed as follows.

Approximately 2 g of each sample material was defatted and dehydrated using acetone and pressurized liquid extraction (Speed Extractor E-916 Büchi, Flawil, Switzerland), according to a method published previously [28]. About (a) 5 mg of the defatted material was used for protein extractions performed with 500 µL of one of the following buffers: Buffer T (TRIS-HCl (1 M, pH 8.2)), buffer TE-50/50 (TRIS-HCl (1 M, pH 8.2)/ethanol, 50/50, *v*/*v*), or buffer TP-50/50 (TRIS-HCl (1 M, pH 8.2)/2-propanol, 50/50, *v*/*v*) for 2 h with constant shaking (1400 rpm) at 60, 80, or 90 °C, respectively [29]. For (b), 50 mg of the defatted samples were extracted with 500 μL of buffer TA-60/40 (TRIS-HCl (1 M, pH 8.2)/ACN, 60/40, *v*/*v*) for 0.5 h with constant shaking (1400 rpm) at 90 °C. After cooling to room temperature, the protein extracts were centrifuged for 10 min at 12,000 rpm. An amount of 200 (TP-50/50, TA-60/40) or 500 μL (TE-50/50) TRIS-HCl (1 M, pH 8.2, buffer T) was added to a 100-μL sample of the protein extracts. The samples which were extracted with buffer T were not diluted. For (a), the protein extracts were additionally reduced with 10 μL DTT solution (200 mM) for 30 min in a thermomixer (60 °C, 1400 rpm) and then alkylated by the addition of 5 μL IAA solution (1 M) for 30 min at room temperature in the dark. Afterwards, 20 μL of Trypsin solution (0.1 μg/mL in 50 mM acetic acid) was added to the protein extracts and incubated at 37 °C for 18 h. The digestion was stopped by the addition of 2 μL concentrated formic acid. Subsequently, the samples were centrifuged for 10 min at 12,000 rpm and, for (a), the supernatants were transferred to 1-mL tapered glass vials and stored at −20 °C. For (b), the supernatants were loaded onto a Chromabond HR-X solid phase extraction column (30 mg/1 mL; Macherey–Nagel, Düren, Germany), which was preconditioned with 1 mL of ACN and 1 mL of water. The samples were washed with 1 mL of water and eluted with 500 µL of 70% 2-propanol in water. The eluates were collected in a 1-mL tapered glass vial, prefilled with 5 µL dimethyl sulfoxide, carefully concentrated in a nitrogen stream, and dissolved in 50 µL of solvent A (see Section 2.2.2).

#### 2.2.2. HPLC-MS/MS-Identification of Peptides for the Nine Legume Species

The experimental procedure comprised sample preparations as described in Section 2.2.1. (a), liquid chromatography—high-resolution mass spectrometry, and data analysis for peptide identification.

##### Liquid Chromatography—High-Resolution Mass Spectrometry

Liquid chromatography was performed on a Dionex UltiMate 3000 RS HPLC from Thermo Scientific (Waltham, MA, USA) equipped with a Nucleosil 100-3 C18 HD (125 × 2 mm; particle size: 3 μm) from Macherey–Nagel (Düren, Germany). The injection volume was 2 μL and the column temperature was set to 40 °C. The mobile phase consisted of solvent A: 3% ACN and 0.1% formic acid in water and solvent B: 90% ACN and 0.1% formic acid in water. The LC run (flow rate: 0.25 mL/min, total time: 52 min) started with 2% B for 3 min, followed by a linear gradient to 60% B in 30 min and another linear gradient to 100% B in 1 min, followed by an isocratic step for 10 min. After switching to 2% B in 1 min, the column was allowed to equilibrate at 2% B for 7 min.

Data for the peptide identification (peak lists of precursor and fragment ions) were obtained by data-dependent high-resolution MS/MS on a maXis UHR-ToF system (Bruker Daltonik, Bremen, Germany) in the positive ESI mode (capillary voltage: 3500 V). The ESI interface setting parameters were 180 °C drying gas temperature and 4 bar ESI nebulizer gas (N_2_) pressure. The mass range of the LC-MS/MS measurements was *m*/*z* 100–1600 with a spectra scan rate of 2 Hz. Selected precursors analyzed more than twice were actively excluded from analysis for 60 s. The collision energy of the quadrupole ranged between 25 and 50 V [28].

##### Data Analysis for Peptide Identification

The peak lists of the data-dependent MS/MS measurements were analyzed with PEAKS Studio 10.0 (Bioinformatics Solutions, Waterloo, ON, Canada) using the enhanced target-decoy method (decoy-fusion) for false discovery rate estimation and result validation [30]. The following parameters were applied for the de novo sequencing: Mass tolerance (precursor and fragment ion tolerance): 0.025 Da, enzyme: Trypsin, no missed cleavages, and fixed modification: Cysteine carbamidomethylation. The peptides identified were searched against the NCBI database (version 7 August 2019) with PEAKS Studio, whereas the taxonomy was restricted to Viridiplantae. The raw data lists of peptides identified per legume species were imported into JMP 15.1.0 (SAS, Heidelberg, Germany). The peptides occurring in only one of the nine legume species were preselected and additionally checked for potential homologies in other species using the online search tool of the NCBI database (BLAST; parameters for database search: Query cover = 100%, percent identity = 100%; accession date: 6 April 2021) with no restriction of the taxonomy.

#### 2.2.3. Synthesis of Peptides

The peptide candidate markers (see Table S1) were synthesized and purified as described previously [29]. The identities of the purified peptides were verified by HPLC-MS/MS. The synthesized peptides were used to select the five most abundant theoretically explainable mass transitions for each peptide and to optimize the multiple reaction monitoring (MRM) parameters (declustering potential, collision energy, and cell exit potential) at the AB Sciex QTrap 5500 (Darmstadt, Germany) using a syringe pump injection. Optimization was automatically performed using the “Compound Optimization” feature of the MS-control software Analyst 1.7.1 (AB Sciex, Darmstadt, Germany).

#### 2.2.4. HPLC-MS/MS-Detection of Marker Peptides for the Nine Legume Species in Emulsion-Type Sausages

The analytical method comprised the preparation of emulsion-type sausages as described in Section 2.2.1. (b) and liquid chromatography—triple quadrupole mass spectrometry.

##### Liquid Chromatography—Triple Quadrupole Mass Spectrometry

Separation of peptides was performed with a Dionex UltiMate 3000 RS HPLC from Thermo Scientific (Waltham, MA, USA) equipped with a Nucleodur C18 Gravity-SB column (50 × 2 mm; particle size 1.8 μm) from Macherey–Nagel (Düren, Germany). The injection volume was 5 μL and the column temperature was set to 50 °C. The mobile phase consisted of solvent A and solvent B (see Section 2.2.2). The LC run (flow rate: 0.5 mL/min) started with 2% B and a gradient to 25% B in 7.9 min. After switching to 100% B in 0.1 min, an isocratic step followed for 4.5 min at 100% B (flow rate: 0.7 mL/min). At the end of the run, the column was allowed to equilibrate at 2% B for 2.5 min at a flow rate of 0.5 mL/min (total time: 15 min).

Peptide detection was carried out on an AB Sciex QTrap 5500 (Darmstadt, Germany) in positive ESI mode using the following parameters: Source temperature: 430 °C, ion spray voltage: 5.5 kV, curtain gas flow: 35 µL/min, and an entrance potential of 10 V. Details of the scheduled MRM method are shown in Table 2.

#### 2.2.5. Statistical Analysis

Calculations were performed with JMP (Version 15.1.0; SAS, Heidelberg, Germany). Repeatability of the method was checked with five independent measurements of seven independent sample preparations of one defatted sausage batch. The unknown sausages (U1–U4) of processing series 2 were treated as samples with unknown concentrations. Their concentrations were calculated by using standard curves (standard sausages) for each legume species. The recoveries were obtained by comparing the calculated and the experimental concentrations as shown in Table 1. Standard box plots were used to visualize the recovery rates. The box plots show the median, quantiles as boxes, and upper and lower ends of the vertical lines extended to 1.5 times the interquartile distance at most. Outliers are displayed as dots.

## 3. Results and Discussion

### 3.1. Determination of Suitable Marker Peptides for Alfalfa, Broad Bean, Chickpea, Lentil, Lupine Blue, Lupine White, Pea, Peanut, and Soy in Plant Material

Two different buffers were used to extract legume proteins (TRIS-HCl (1 M, pH 8.2)) [25] or grain proteins (TRIS-HCl (1 M, pH 8.2)/ethanol, 50:50, *v*/*v*) [28] in meat products in previous investigations. Furthermore, it is known from the scientific literature that the addition of 50% 2-propanol to the extraction buffer increases the protein extraction yield in defatted soybean meal [38]. Consequently, the protein extractions of the plant materials were performed applying these three different buffers (buffer T: TRIS-HCl (1 M, pH 8.2); buffer TE-50/50: TRIS-HCl (1 M, pH 8.2)/ethanol, 50/50, *v*/*v*; buffer TP-50/50: TRIS-HCl (1 M, pH 8.2)/2-propanol, 50/50, *v*/*v*) at temperatures of 60, 80, and 90 °C, each for the identification of peptides by high-resolution mass spectrometry. Compared to 60 °C (=100%), the higher extraction temperature increased the number of peptides detected obtained by means of *de novo* sequencing and a subsequent database search (NCBI). A total of 1392 different peptides with 5–45 amino acids, *m*/*z* 336–1273, and an ion charge of 2–5 were detected for all buffer systems tested: Buffer T (80 °C = 99%, 90 °C = 107%), buffer TE-50/50 (80 °C = 151%, 90 °C = 141%), and buffer TP-50/50 (80 °C = 138%, 90 °C = 151%). Comparing the extraction efficiency of the three buffers tested at 90 °C, the most peptides were obtained for buffer T-50/50 (1019 peptides), followed by buffer TP-50/50 (765 peptides) and buffer TE-50/50 (288 peptides).

The peptides obtained by means of database search (NCBI; alfalfa: 109; broad bean: 223; chickpea: 176; lentil: 206; lupine blue: 143; lupine white: 152; pea: 315; peanut: 195, and soy: 240) were statistically evaluated, preselecting peptides only detected in a single legume species (alfalfa: 70; broad bean: 131; chickpea: 128; lentil: 91; lupine blue: 33; lupine white: 42; pea: 188; peanut: 192, and soy: 233; in total: 1108 peptides). No universal marker occurring in all nine (or at least eight) legume species was detected and only one peptide each was detected common for six or seven species, respectively. The majority of the peanut and soy peptides (98% and 97%, respectively) were not detected in the other legume species.

After considering further preselecting criteria (6–20 amino acids; no cysteine) [40], the preselected peptides and the tryptic (no missed cleavages), heat-stable, and specific marker peptides for lupine blue, pea, peanut, and soy reported in the scientific literature (see Table S1) were searched against the NCBI database using the online BLAST algorithm (https://blast.ncbi.nlm.nih.gov/BLAST.cgi, accessed on 6 April 2021). The resulting peptides (potential candidates) for alfalfa, broad bean, chickpea, lentil, and lupine white with entries for only one of these legume species and no entries for relevant matrices such as meat species or spices were verified by measuring the tryptic digests of the legume flours applying an MRM method with non-optimized MS/MS-parameters. In this approach, the five most abundant fragment ions with *m*/*z* >250 determined by previous measurements in the enhanced product ion scan mode were used as mass transitions.

The most intense peptides for alfalfa, broad bean, chickpea, lentil, and lupine white according to the MRM measurements (non-optimized MS/MS parameters) and the peptides from the scientific literature were synthesized (candidate markers; see Table S1). The synthesized peptides were used to select the five most abundant theoretically explainable mass transitions (*m*/*z* > 250 Da) for each peptide and optimize the MRM parameters (see Table 2). Three peptides (8–18 amino acids) for each legume species were finally chosen as peptide markers from the group of the candidate marker peptides due to their highest intensities in spiked sausages (test sausage T4, see Table 1) applying the optimized MRM method (see Section 3.2.). The final selected peptides QQEQQLEGELEK [25], NTLEATFNTR [25,31], and ISSVNSLTLPILR [25] for lupine blue, NPYHFSSQR [31] for lupine white, ELTFPGSVQEINR, LSSGDVFVIPAGHPVAVK, and LTPGDVFVIPAGHPVAVR for pea [25], GTGNLELVAVR [32,33,34,35,36], FNLAGNHEQEFLR [32,33,37,38], and WLGLSAEYGNLYR [32,34] for peanut, and SQSDNFEYVSFK [23,34,36], EAFGVNMQIVR [25,34,38,39], and FYLAGNQEQEFLK [22,23,25,36] for soy have been reported previously in the scientific literature. The identities of each of the 14 marker peptides for alfalfa, broad bean, chickpea, and lentil, as well as lupine white 2 and 3, which have not been reported in the literature until now, were confirmed by spiking in a tryptic digest of an emulsion-type sausage (test sausage T4). This was particularly important for lentil 1 (VILEDQEQEPQHR) since the amino acid sequence suggested initially was the isomeric peptide VLLEDQEQEPQHR (NCBI accession Q84UI0). This and the other two isomeric peptides VLIEDQEQEPQHR and VIIEDQEQEPQHR could be excluded due to different retention times (t_R_s). The legume marker peptides used originated from at least two different target proteins for each legume species, to minimize the general risk of adverse analytical effects due to different technological treatments of the samples (see Table S2) [40].

According to the BLAST database search, the lentil marker peptide FFEVTPEK showed homologies to *Lens nigricans*. All three peanut marker peptides showed homologies to *Arachis ipaensis* and *Arachis duranensis*, all three peptides of soy showed homologies to *Glycine soja*, and all three marker peptides of alfalfa showed homologies to *Medicago truncatula* (see Table S2). However, all these homologies were restricted to the genus of the respective legume species.

In addition, the uniqueness of the final marker peptides was checked experimentally by analyzing the nine legume flours and emulsion-type sausages without the addition of any type of legume (blank value) in the MRM mode. Furthermore, seven groups containing a total of 46 common possible ingredients of meat products and twelve different commercial spice mixtures (see Table S3) were analyzed. This approach was essential, particularly because the matrices mentioned can contain peptides with mass transitions nearly identical to those of the final marker peptides, especially using low-resolution MS methods, which, consequently, might lead to false-positive results [41]. In accordance with the NCBI database search (see Table S2), lupine blue 2 was also detected in the pure seeds of lupine white. None of the final marker peptides were detected in the meat matrix or the ingredient groups/spice mixture analyzed with the exception of the three alfalfa peptides, which were detected in fenugreek (*Trigonella foenum-graecum*), a common ingredient of curry spice mixtures [42]. The addition of curry spice mixture was compared to the addition of alfalfa flour in emulsion-type sausages in different concentrations (0.05, 0.5, and 2.5%). It was shown that the intensities of the alfalfa peptides adding a low amount of 0.05% alfalfa flour were comparable to an unusual high quantity of 2.5% curry spice mixture added.

### 3.2. Optimization of the Conditions of Protein Extraction and Tryptic Digestion in Meat Products with Added Legume Protein Flour

After the selection of suitable peptide markers for the detection of alfalfa, broad bean, chickpea, lentil, lupine (blue and white), pea, peanut, and soy, the analytical method was optimized according to the conditions of protein extraction, tryptic digestion, and purification by solid phase extraction. The optimization was performed with emulsion-type sausages with 0.1% legume protein, each (test sausage T4; see Table 1). Within these experiments, the five mass transitions selected for each marker peptide (see Section 3.1.) were restricted to the three most abundant mass transitions in the meat matrix.

It became apparent from the results of the experiments to identify the marker peptides (without meat matrix) that a higher temperature supports the extraction of legume proteins (see Section 3.1.), resulting in the highest number of legume peptides identified at a temperature of 90 °C. Analogous to these experiments, different protein extraction temperatures (60, 80, and 90 °C) applying the buffers T, TP-50/50, and TE-50/50 were tested for the emulsion-type sausages. Compared to 60 °C (=100%), higher extraction temperatures (N = 3) led to higher normalized mean peak areas showing the highest mean values for 90 °C (buffer T: 80 °C = 128%, 90 °C = 167%; buffer TP-50/50: 80 °C = 143%, 90 °C = 171%; buffer TE-50/50: 80 °C = 180%, 90 °C = 288%).

Comparing the peak areas using buffer T, TE-50/50, and TP-50/50 at an extraction temperature of 90 °C showed the highest values for buffer TP-50/50 (see Table 3). Subsequently, further experiments were carried out using different contents of 2-propanol (TP-60/40, TP-70/30) showing the best intermediate results for TP-60/40. As previous investigations showed that the extraction of proteins from the meat matrix was increased by using ACN in the extraction buffer (buffer TA: TRIS-HCl (1 M, pH 8.2)/ACN)) [29], the buffers TA-50/50, TA-60/40, and TA-70/30 were also tested. The mean peak areas (N = 3) of the legume peptides (extraction temperature: 90 °C) were individually normalized to the highest peak area (=100%; Table 3). Nineteen of 27 peptides showed maximum normalized mean peak areas using the buffer TA-60/40. Seven peptides showed normalized mean peak areas in the range of 91–98% and only one peptide (peanut 3) at 82%. Furthermore, the highest mean peak area (calculated from the normalized mean peak areas of all peptides) was observed for buffer TA-60/40 (98%), compared to the other buffers showing means in the range of 31 (buffer T) to 88% (buffer TA-50/50).

In a next step, different extraction times were tested applying buffer TA-60/40 at 90 °C. The highest mean peak area (calculated from the normalized mean peak areas of all peptides) was observed for the extraction time of 30 min (95%). At this duration, the most peptide markers (13 of 27) showed their maximum peak areas. However, the means of the normalized mean peak areas did not show any major variations and ranged between 85 (90 min) and 91% (60 and 150 min) for the other extraction times. Based on these findings, the extraction time was set to 30 min, also showing the advantage of reducing the sample preparation time, and, consequently, enabling a higher sample throughput.

It was shown in a previous study [25] that the use of DTT and IAA, despite the occurrence of disulfide bonds in the target proteins of lupine blue and soy, did not improve the efficiency of the tryptic digestion. These findings were confirmed for these target proteins. Furthermore, the addition of DTT and IAA did not improve the digestion yield of the other legume proteins included in this study. The solid phase extraction elution mixture used in the study mentioned [25] (80% ACN in water) was tested in comparison with several other elution mixtures (each 90, 80, and 70% of ACN, ethanol and 2-propanol in water). It was shown that the use of 70% 2-propanol in water gave the best results for recovery.

### 3.3. Detection of Legume Peptide Markers and Quantification of Meat Substitution by Legume Proteins in Emulsion-Type Sausages

**Figure 1:**
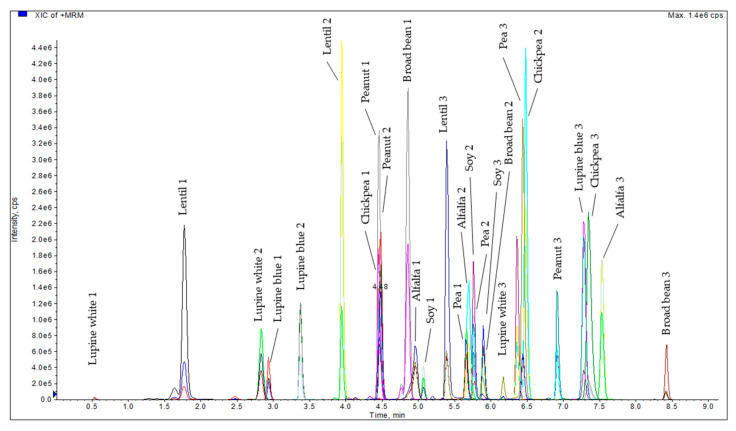
Chromatogram of the synthesized legume marker peptides (7 ng/µL) in a spiked control emulsion-type sausage.

A UHPLC method (see Section 2.2.4.) was developed and the gradient of the method was optimized by measuring a control sausage spiked with the synthesized marker peptides (see Table 2) at a concentration level of about 7 ng/µL each (Figure 1).

The production of meat products for this study focused on emulsion-type sausages due to their homogeneity and the technological experience with the addition of plant proteins [25,28]. All legume flours were added at the same protein levels (see Table 1) for the production of the four batches of processing series 1 (test sausages, T1–T4). The sausages of series 1 were used for method development (see Section 3.2.), the determination of the limit of detection (LOD), and to test the repeatability of the method. Three marker peptides were determined for each legume species (see Table 2) for the clear evidence of legume proteins in emulsion-type sausages. The three most abundant mass transitions of each marker peptide must have a signal-to-noise ratio (S/N) equal to or greater than 3 according to the commonly used definition of the LOD for a reliable detection [43]. The LODs were ≤0.001% for broad bean and soy, ≤0.004% for chickpea, lupine blue, pea, and peanut, and ≤0.01% for alfalfa, lentil, and lupine white (related to the protein content of each legume species in the emulsion-type sausage).

The emulsion-type sausage with the lowest level in which all legume species were detectable (T3; 0.01% added legume protein) was used for the determination of the repeatability of the analytical method. A sample of the defatted material of this batch was split into 14 aliquots. Two technical assistants prepared seven aliquots, each, on five different days. Each of the samples was measured on the following day. Accordingly, a total of 70 samples were prepared and measured. The coefficients of variation (CVs) of the peak areas were calculated in order to determine the repeatability. The CVs were below 20% (N = 70) for all marker peptides of broad bean, chickpea, pea, and soy (see Table S4). Furthermore, the CVs of at least one marker peptide of the five remaining legume species were ≤20%. The CVs of alfalfa 1, chickpea 1, lupine blue 1, peanut 2, and soy 3 were even ≤10%. However, the CVs of eight marker peptides (alfalfa 2, alfalfa 3, lentil 1, lentil 3, lupine blue 2, lupine blue 3, lupine white 1, and peanut 3) were >20%. The CVs of the ratios of the peak areas of the lowest to the highest intense mass transitions (as shown in Table 2) were all ≤20%, with the exception of lupine blue 3 (24%). Furthermore, the CVs of the mass transition ratios of 22 of the 27 marker peptides were ≤10%. The standard deviations of the retention times (N = 70; see Table 2) were low and ranged between ±0.01 and ±0.06 min.

A protein content in the pork of 19.5% was assumed for processing series 2 [4]. The amounts of legume flours added were calculated in such a way that the reduced amount of meat protein was substituted by the same amount of legume protein for each concentration level (as shown in Table 1) with added legume protein (standard sausages S1–S9: 0.1, 0.4, 0.7, 1.0, 1.3, 1.6, 1.9, 2.2, and 2.5%; unknown sausages U1–U4: 0.0, 0.85, 1.75, and 2.35%) to keep the total protein content of the sausages at a constant level (see Table 1). To the best of our knowledge, a threshold for the meat substitution in meat products by plant proteins not belonging to the 14 EU main allergens does not exist. In the United Kingdom, however, a threshold of 1% (*w*/*w*) undeclared meat in comminuted meat was established [44], and the UK Food Standards Agency (FSA) recommends investigation of the causes of contamination between 0.1% and 1% (*w*/*w*) [45]. Based on the lower limit recommended by FSA and on the results of the determination of the LOD using the sausages of processing series 1, the lowest limit of the meat substitution was set at 0.1% (0.01% protein content of each legume species).

The standard sausages (S1–S9) were used to obtain standard curves for the quantification of each legume species. The unknown sausages (U1–U4) were produced under the same conditions as the standard and were produced in such a manner that 2–3 legume species in each batch were missing (see Table 1). With the exception of lupine blue and white, no false-positive results were obtained from the unknown sausages. The peptides of lupine blue were detected in U4 (no lupine blue added) and of lupine white were detected in U1 (no lupine white added). Consequently, the lupine flours used for processing series 2 were analyzed, showing that neither of them was entirely pure for the respective lupine species. However, a correlation between the peak areas of lupine peptides (lupine blue 2, lupine white 1, and lupine white 2) and the total meat substitution by lupine protein (blue and white) was observed (*R*^2^-values: 0.91–0.99).

**Figure 2:**
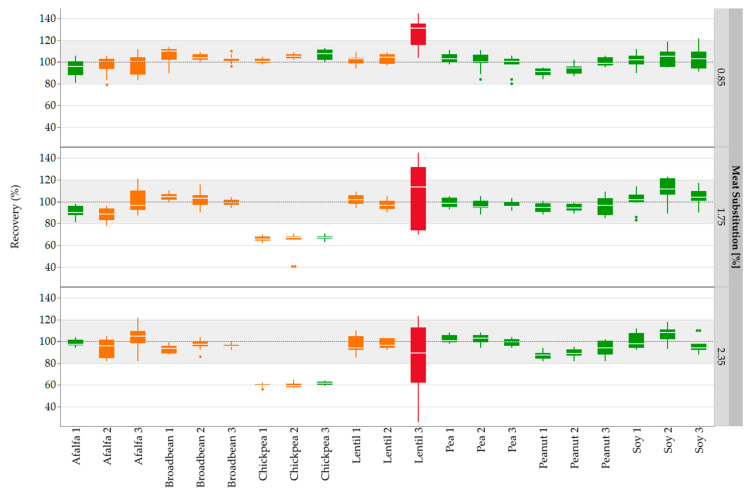
Recovery rates of the legume marker peptides in emulsion-type sausages (0.85, 1.75, and 2.35% meat substitution) quantified with standard sausages (0.1–2.5% meat substitution). All three concentration levels (0.85, 1.75, and 2.35%) were measured in duplicate from two different sausage samples and three independent sample preparations: Box plots are from twelve measurements. The gray areas represent the accepted range of 80–120%. Correlation coefficients (*R*^2^) were mapped with the use of a color code: Red: *R*^2^ = 0.87; Orange: *R*^2^ = 0.95–0.97; Green: *R*^2^ > 0.97.

Quantifications were performed only for alfalfa, broad bean, chickpea, lentil, pea, peanut, and soy due to the impurities of the lupine flours used in processing series 2. The mean correlation coefficients (*R*^2^) of 20 out of a total of 21 peptides ranged between 0.95 (alfalfa 2) and 0.99 (all pea markers and soy 3) (N = 12). Only the *R*^2^ value of lentil 3 was noticeably lower (0.87). The recovery rates for the unknown sausages were calculated and a range of ±20% was accepted [46]. Most recovery rates were within the accepted range of 80–120% (Figure 2). Despite high *R*^2^ values (0.97–0.98) for the chickpea peptides, lower recovery rates (<80%) were obtained for chickpea in the unknown sausages with 1.75% and 2.35% meat substitution. However, the variations of the recovery rates for the three chickpea peptides were low, indicating that a systematic error probably occurred during the production of the sausages U3 and U4. Furthermore, the recovery rates for lentil 3 showed high variations in all three concentrations.

## 4. Conclusions

The analytical mass spectrometric methods existing currently for the detection of legume proteins in meat products (and also in other foodstuffs) were primarily restricted to the allergenic legume species lupine, peanut, soy, and, to a lesser extent, pea. However, methods for the detection of the legume species alfalfa, broad bean, chickpea, and lentil and, consequently, specific marker peptides were lacking. Since these legume species not belonging to the 14 EU main allergens are possibly more relevant as potential foreign proteins due to a low allergenic risk, twelve new specific marker peptides were identified and included in an UHPLC-MS/MS method. The latter enabled, on the one hand, the simultaneous detection of nine legume species using an optimized extraction protocol and applying a short LC/MS measurement time (15 min) allowing a high throughput. On the other hand, the amounts of the legume proteins added could be quantified with the help of a matrix calibration and, consequently, a differentiation of a conscious use of legume protein for meat substitution and inadvertently transferred cross-contaminations (traces) is possible. Since the developed UHPLC-MS/MS method was suitable to detect the food fraud of an undeclared meat substitution by legume proteins, it seems to be a promising analytical tool for food control authorities. Therefore, the introduction of the method into the German §64 LFGB working group “Mass Spectrometric Protein Analysis” [47], with the aim of including the validated method in the “Official Collection of Methods of Analysis and Sampling” will be pursued. Furthermore, the presented method and especially the new marker peptides represent a solid basis for the further development to an allergen detection method also including non-priority legume allergens such as chickpea, lentil, lupine, and pea [48].

## Figures and Tables

**Table 1:** Formulations and batches of emulsion-type sausages with different concentrations of legume flours for processing series 1 (test sausages (T1–T4: 0.001, 0.004, 0.01, and 0.1% legume protein, each) and processing series 2 (standard (S1–S9) and unknown (U1–U4) samples); LFM = legume flour mixture; C = control.

	Processing Series 1					Processing Series 2													
	C1	Test Sausages				C2	Standard Sausages									Unknown Sausages			
	0	T1	T2	T3	T4	0	S1	S2	S3	S4	S5	S6	S7	S8	S9	U1	U2	U3	U4
**Formulations (%)**																			
Pork	54	53.98	53.9	53.8	51.6	50	44.15	44.15	44.15	44.15	44.15	44.15	44.15	44.15	44.15	45.05	44.63	44.50	44.35
Back fat	24	24	24	24	24	24	24	24	24	24	24	24	24	24	24	24	24	24	24
Curing salt	1.8	1.8	1.8	1.8	1.8	1.8	1.8	1.8	1.8	1.8	1.8	1.8	1.8	1.8	1.8	1.8	1.8	1.8	1.8
Phosphate	0.2	0.2	0.2	0.2	0.2	0.2	0.2	0.2	0.2	0.2	0.2	0.2	0.2	0.2	0.2	0.2	0.2	0.2	0.2
Ice	20	20	20	20	20	24	27.30	26.89	26.75	26.36	26.31	27.00	26.45	26.99	27.31	26.50	26.24	26.64	26.96
LFM	-	0.02	0.1	0.2	2.4	-	2.55	2.96	3.10	3.49	3.54	2.85	3.40	2.86	2.54	2.45	3.14	2.86	2.69
**Legume Flour (%)**																			
Alfalfa	-	0.003	0.011	0.03	0.27	-	0.03	0.10	0.07	0.26	0.34	0.42	0.50	0.58	0.66	0.62	-	0.22	0.46
Broad bean	-	0.004	0.016	0.04	0.40	-	0.15	0.27	0.10	0.50	0.62	0.73	0.85	0.96	0.04	0.67	0.91	-	0.33
Chickpea	-	0.005	0.018	0.04	0.46	-	0.33	0.47	0.13	0.75	0.89	0.21	1.17	0.05	0.19	-	0.40	0.82	1.10
Lentil	-	0.004	0.016	0.04	0.40	-	0.37	0.47	0.16	0.69	0.80	0.91	0.04	0.15	0.26	0.31	0.64	0.86	-
Lupine blue	-	0.002	0.009	0.02	0.23	-	0.29	0.35	0.19	0.49	0.55	0.02	0.09	0.16	0.22	0.19	0.39	0.52	-
Lupine white	-	0.002	0.010	0.02	0.25	-	0.40	0.47	0.21	0.62	0.02	0.10	0.17	0.25	0.32	-	0.21	0.44	0.59
Pea	-	0.001	0.005	0.01	0.13	-	0.23	0.27	0.24	0.01	0.05	0.09	0.12	0.16	0.20	0.29	-	0.10	0.21
Peanut	-	0.002	0.008	0.02	0.21	-	0.47	0.53	0.01	0.09	0.15	0.21	0.28	0.34	0.41	0.37	0.50	-	0.18
Soy	-	0.001	0.005	0.01	0.12	-	0.29	0.01	0.04	0.08	0.12	0.15	0.18	0.22	0.25	-	0.10	0.20	0.27
**Meat Substitution (%)**																			
Alfalfa	-	-	-	-	-	-	0.10	0.40	0.70	1.00	1.30	1.60	1.90	2.20	2.50	2.35	-	0.85	1.75
Broad bean	-	-	-	-	-	-	0.40	0.70	1.00	1.30	1.60	1.90	2.20	2.50	0.10	1.75	2.35	-	0.85
Chickpea	-	-	-	-	-	-	0.70	1.00	1.30	1.60	1.90	2.20	2.50	0.10	0.40	-	0.85	1.75	2.35
Lentil	-	-	-	-	-	-	1.00	1.30	1.60	1.90	2.20	2.50	0.10	0.40	0.70	0.85	1.75	2.35	-
Lupine blue	-	-	-	-	-	-	1.30	1.60	1.90	2.20	2.50	0.10	0.40	0.70	1.00	0.85	1.75	2.35	-
Lupine white	-	-	-	-	-	-	1.60	1.90	2.20	2.50	0.10	0.40	0.70	1.00	1.30	-	0.85	1.75	2.35
Pea	-	-	-	-	-	-	1.90	2.20	2.50	0.10	0.40	0.70	1.00	1.30	1.60	2.35	-	0.85	1.75
Peanut	-	-	-	-	-	-	2.20	2.50	0.10	0.40	0.70	1.00	1.30	1.60	1.90	1.75	2.35	-	0.85
Soy	-	-	-	-	-	-	2.50	0.10	0.40	0.70	1.00	1.30	1.60	1.90	2.20	-	0.85	1.75	2.35
Total	-	-	-	-	-	-	11.70	11.70	11.70	11.70	11.70	11.70	11.70	11.70	11.70	9.90	10.75	11.00	11.30

**Table 2:** Parameters of the scheduled MRM method (MRM detection window 40 s; CE = collision energy; CXP = cell exit potential; DP = declustering potential). The product ions are listed in decreasing intensity.

Marker Peptide		t_R_ [Min]	DP [V]	*m*/*z* (Charge State)	Product Ions	CE [V]	CXP [V]
Alfalfa 1	VEGGLSIMSPPER	4.91 ± 0.02	71	686.4 (+2)	498.3 (y4), 585.3 (y5), 716.3 (y6)	27/29/31	26/26/24
Alfalfa 2	FNLEAGDIMR	5.75 ± 0.02	56	583.3 (+2)	662.3 (y6), 419.2 (y3), 591.3 (y5)	29/39/27	32/24/30
Alfalfa 3	ISDVNSLTLPILR	7.60 ± 0.01	60	720.9 (+2)	498.3 (y4), 712.5 (y6), 316.2 (b3)	35/35/35	36/36/36
Broad bean 1	EDVLSLAPK	4.77 ± 0.02	36	486.3 (+2)	515.3 (y5), 315.2 (y3), 628.4 (y6)	23/21/25	24/16/32
Broad bean 2	FNLEEGDLIR	5.90 ± 0.01	16	603.3 (+2)	702.4 (y6), 401.3 (y3), 573.3 (y5)	31/43/31	28/22/26
Broad bean 3	LSPGDVLVIPAGYPVAIK	8.47 ± 0.01	16	603.7 (+3)	458.3 (y9^2+^), 527.4 (y5), 514.8 (y10^2+^)	21/35/15	24/36/36
Chickpea 1	GGLSFISPSEK	4.44 ± 0.02	11	561.3 (+2)	547.3 (y5), 460.2 (y4), 660.4 (y6)	27/37/27	28/30/30
Chickpea 2	IVDLAIPINTPAK	6.53 ± 0.01	26	682.9 (+2)	740.4 (y7), 328.2 (b3), 625.4 (b6)	29/35/25	42/22/30
Chickpea 3	SSNPFTFLVPPR	7.46 ± 0.01	91	681.8 (+2)	369.2 (y3), 468.3 (y4), 581.4 (y5)	25/33/33	24/22/32
Lentil 1	VILEDQEQEPQHR	1.87 ± 0.03	31	540.9 (+3)	704.8 (y11^2+^), 648.3 (y10^2+^), 470.2 (y11^3+^)	23/21/23	40/42/24
Lentil 2	FFEVTPEK	3.93 ± 0.01	66	498.8 (+2)	702.4 (y6), 373.2 (y3), 573.3 (y5)	21/35/25	34/28/32
Lentil 3	VVDFVISLNRPGK	5.41 ± 0.03	101	481.9 (+3)	623.4 (y11^2+^), 301.2 (y3), 884.5 (y8)	19/37/27	44/20/40
Lupine blue 1	QQEQQLEGELEK [25]	2.48 ± 0.02	36	729.9 (+2)	389.2 (y3), 386.2 (b3), 575.3 (y5)	37/35/33	29/18/28
Lupine blue 2	NTLEATFNTR [25,31]	3.35 ± 0.01	31	583.8 (+2)	838.4 (y7), 709.4 (y6), 458.2 (b4)	31/31/21	50/40/26
Lupine blue 3	ISSVNSLTLPILR [25]	7.11 ± 0.01	45	706.9 (+2)	498.3 (y4), 712.5 (y6), 359.2 (a4)	33/31/35	22/30/24
Lupine white 1	NPYHFSSQR [31]	0.49 ± 0.06	21	379.2 (+3)	341.2 (y8^3+^), 373.7 (b6^2+^), 390.2 (y3)	17/13/23	20/24/20
Lupine white 2	DKPSQSGPFNLR	2.83 ± 0.02	46	449.2 (+3)	323.7 (y5^2+^), 646.4 (y5), 549.3 (y4)	19/21/27	16/30/40
Lupine white 3	AVNELTFPGSAEDIER	5.90 ± 0.01	46	583.3 (+3)	487.2 (y9^2+^), 560.8 (y10^2+^), 417.2 (y3)	21/15/41	22/40/22
Pea 1	ELTFPGSVQEINR [25]	5.68 ± 0.02	41	745.4 (+2)	999.5 (y9), 500.3 (y9^2+^), 491.3 (b4)	37/37/29	48/28/26
Pea 2	LSSGDVFVIPAGHPVAVK [25]	5.72 ± 0.02	120	598.3 (+3)	438.3 (y9^2+^), 513.3 (y5), 363.2 (y11^3+^)	27/37/43	34/24/24
Pea 3	LTPGDVFVIPAGHPVAVR [25]	6.57 ± 0.01	36	615.7 (+3)	544.3 (y16^3+^), 541.3 (y5), 452.3 (y9^2+^)	21/35/33	28/38/14
Peanut 1	GTGNLELVAVR [32,33,34,35,36]	4.45 ± 0.02	96	564.8 (+2)	345.2 (y3), 557.4 (y5), 686.4 (y6)	29/33/31	24/28/38
Peanut 2	FNLAGNHEQEFLR [32,33,37,38]	4.45 ± 0.02	61	525.6 (+3)	262.1 (b2), 657.3 (y11^2+^), 600.8 (y10^2+^)	23/23/23	14/40/16
Peanut 3	WLGLSAEYGNLYR [32,34]	7.02 ± 0.01	16	771.4 (+2)	272.2 (a2), 300.2 (b2), 357.2 (b3)	39/35/39	14/18/18
Soy 1	SQSDNFEYVSFK [23,34,36]	5.02 ± 0.04	31	725.8 (+2)	381.2 (y3), 643.3 (y5), 1235.6 (y10)	35/35/29	26/46/52
Soy 2	EAFGVNMQIVR [25,34,38,39]	5.77 ± 0.02	61	632.3 (+2)	760.4(y6), 387.3 (y3), 532.3 (y9^2+^)	29/29/27	38/22/34
Soy 3	FYLAGNQEQEFLK [22,23,25,36]	6.00 ± 0.01	36	793.9 (+2)	311.1 (b2), 424.2 (b3), 638.8 (y11^2+^)	41/35/33	18/26/38

**Table 3:** Mean peak areas (N = 3, each) of the legume marker peptides using different buffers at 90 °C and 2 h extraction time (most intense mass transitions as shown in Table 2; expressed as a percentage of the highest intensity (=100%; marked in bold); T = TRIS-HCl [1 M, pH 8.2], TE = TRIS-HCl [1 M, pH 8.2]/ethanol, TP = TRIS-HCl [1M, pH 8.2]/2-propanol, TA = TRIS-HCl [1 M, pH 8.2]/ACN) and using different extraction times. Extraction was performed with the buffer TA-60/40 at 90 °C for studies on extraction times.

	Extraction Buffer								Time (Min)					
	T	TE	TP			TA								
	100	50/50	50/50	60/40	70/30	50/50	60/40	70/30	30	60	90	120	150	180
Alfalfa 1	79	88	72	54	58	65	92	**100**	69	86	97	42	**100**	44
Alfalfa 2	14	35	41	52	45	67	**100**	75	93	89	66	92	91	**100**
Alfalfa 3	3	57	59	58	41	100	98	42	**100**	90	39	97	97	84
Broad bean 1	30	90	72	76	49	72	**100**	88	**100**	96	83	94	96	98
Broad bean 2	26	76	63	72	51	74	**100**	96	**100**	96	84	87	85	92
Broad bean 3	94	76	48	55	56	90	**100**	92	93	96	97	93	96	**100**
Chickpea 1	47	36	62	71	65	87	98	**100**	91	88	88	**100**	95	92
Chickpea 2	16	22	72	72	38	87	**100**	82	100	86	88	89	97	81
Chickpea 3	27	23	60	47	27	**100**	**100**	77	97	88	88	89	**100**	95
Lentil 1	12	20	76	74	39	87	**100**	79	79	88	90	94	**100**	96
Lentil 2	10	18	63	65	36	87	**100**	80	83	90	89	96	**100**	98
Lentil 3	7	14	36	67	36	83	**100**	78	92	89	92	**100**	**100**	98
Lupine blue 1	38	42	74	84	77	**100**	98	98	**100**	90	75	83	87	86
Lupine blue 2	19	17	45	54	40	92	**100**	89	**100**	**100**	70	83	81	80
Lupine blue 3	15	6	37	48	36	82	**100**	90	**100**	88	54	96	96	**100**
Lupine white 1	38	42	74	84	77	**100**	98	98	99	95	95	**100**	94	89
Lupine white 2	19	17	45	54	40	92	**100**	89	89	95	97	98	**100**	95
Lupine white 3	15	11	37	48	35	82	**100**	90	80	88	90	95	**100**	96
Pea 1	21	35	85	84	72	83	**100**	85	89	**100**	**100**	93	80	95
Pea 2	28	24	66	73	68	85	**100**	89	94	**100**	**100**	90	94	91
Pea 3	22	21	54	62	58	86	**100**	85	99	**100**	94	93	95	95
Peanut 1	69	43	71	70	61	98	**100**	87	**100**	86	81	71	61	56
Peanut 2	70	24	57	49	39	**100**	91	60	**100**	90	94	82	91	92
Peanut 3	73	59	81	71	72	**100**	82	59	**100**	83	85	72	79	80
Soy 1	29	29	67	67	58	96	**100**	93	**100**	80	77	77	73	68
Soy 2	6	16	52	43	27	**100**	98	68	**100**	95	87	94	88	77
Soy 3	28	33	77	60	53	95	**100**	86	**100**	92	89	92	86	75
**Mean**	31	35	61	64	50	88	**98**	83	**95**	91	85	89	91	87
**Maxima (N)**	0	0	0	0	0	7	**19**	2	**13**	4	2	3	7	3

## Data Availability

Data is contained within the article or Supplementary Material.

## Supplementary Materials

The following materials are available online at https://www.mdpi.com/article/10.3390/foods10050947/s1, Table S1: Synthesized peptides (candidate markers) for the nine legume species and corresponding target proteins (marker peptides in bold were selected for the final method); Table S2: Marker peptides for alfalfa, broad bean, chickpea, lentil, lupine blue, lupine white, pea, peanut, and soy and their homologies (NCBI online search tool BLAST, parameters for database search: query cover = 100%, percent identity = 100%; without bacteria); * Target proteins refer to Medicago truncatula; Table S3: Groups of possible ingredients for the production of sausages and commercial spice mixtures which were tested regarding cross-reactivity with the legume marker peptides analyzed; Table S4: Normalized peak areas of legume marker peptides expressed as a percentage of the most intense marker for a species (= 100%; marked in bold), CVs of the peak areas, and CVs of the ratio of peak area of the lowest to the highest intense mass transition as shown in Table 2 (N = 70, each).
