# Supplementary material for: A UHPLC-MS/MS Method for the Detection of Meat Substitution by Nine Legume Species in Emulsion-Type Sausages
Source: Foods. 2021 Apr 26;10(5):947. doi: 10.3390/foods10050947 (PMC8146705; doi:10.3390/foods10050947)
Supplement: Supplementary file 1 [file foods-10-00947-s001.zip › foods-1157275-sul.pdf]

Supplementary Table S1. Synthesized peptide candidate markers for the nine legume species and corresponding target proteins (marker peptides in bold were selected for the final method)

Legume species	Target protein	Peptide sequence	References
Alfalfa	legumin A	FYLAGNQEQEFLQYQQQQVR	
		IESEGGIIETWNPNNR	
		TITSFDLPALR	
		**VEGGLSIMSPPER**	
	legumin K	**ISDVNSLTLPILR**	
	sucrose-binding protein	**FNLEAGDIMR**	
	protein SLE1	LGETVIPGGTGGK	
	provicilin	LPAGTLGYLANR	
		SNLFQNLK	
		VLIEENEQEPQHR	
broad bean	convicilin	GVSSELEPFNLR	
	legumin	IVNSQGNPVFDDK	
	putative sucrose binding protein	**EDVLSLAPK**	
		ELETVLDEQQK	
		**FNLEEGDLIR**	
	vicilin	AILTVLLPNDR	
		LPAGTIGYLVNR	
		**LSPGDVLVIPAGYPVAIK**	
		NILEASFNTDYK	
		STSSESEPFNLR	
chickpea	legumin-like	FNSPFSFLVPPR	
		**GGLSFISPSEK**	
		**SSNPFTFLVPPR**	
	provicilin-like	GNFELVGLR	
		ILLEEPQQR	
		**IVDLAIPINTPAK**	
	seed biotin-containing protein SBP65	VIVVGEGDVEEK	
lentil	Allergen Len c 1.0101	NILEAAFNTEYEEIEK	
	Allergen Len c 1.0102	AVLTVLNSNDR	
		SILEAAFNTDYEEIEK	
		**VLLEDQEQEPQHR**	
	Convicilin	FEAFDLSANR	
		**FFEVTPEK**	
		VVEEEEGEWR	
		**VVDFVISLNRPGK**	
		VLLEEQENEPHQR	
	Non-specific lipid-transfer protein 6 (LTP6)	LLAAANTTPDR	
	vicilin type C	SDQDNPFIFESK	
lupine blue	Conglutin alpha 1	TLTSLDFPILR	[1]
	Conglutin beta	**NTLEATFNTR**	[1]
	Conglutin delta 3	**QQEQQLEGELEK**	[1]
	Legumin J-like	**ISSVNSLTLPILR**	[1]
lupine white	Conglutin beta 1	**DKPSQSGPFNLR**	
	Conglutin beta 2	**AVNELTFPGSAEDIER**	
		DKPSDSGPFNLR	
	Conglutin beta 5	**NPYHFSSQR**	[2]
	putative 11-S seed storage protein	HNIGESTSPDAYNPQAGR	
pea	Convicilin	**ELTFPGSVQEINR**	[1]
	Provicilin	**LTPGDVFVIPAGHPVAVR**	[1]
	Vicilin	**LSSGDVFVIPAGHPVAVK**	[1]
peanut	Allergen Ara h 1	DLAFPGSGEQVEK	[3], [4], [5], [6], [7], [8], [9], [10], [11]

		**GTGNLELVAVR**	[5], [12], [13], [14]
		VLLEENAGGEQEER	[3], [4], [5], [8], [9], [10], [15], [16], [17]
	Allergen Ara h 3/ h 4	**FNLAGNHEQEFLR**	[12], [13], [17], [18]
		**WLGLSAEYGNLYR**	[5], [12]
soy	beta-conglycinin alpha-subunit	LITLAIPVNKPGR	[18], [19], [20], [21]
	beta-conglycinin storage protein	DSYNLQSGDALR	[13]
		QQQEEQPLEVR	[13], [21]
	CG4 beta-conglycinin	DSYNLHPGDAQR	[13]
	glycinin G1	VFDGELQEGR	[2], [3], [4], [20], [21], [22]
	Glycinin G1 precursor	**FYLAGNQEQEFLK**	[1], [15], [23], [24],
		**SQSDNFEYVSFK**	[5], [15], [24]
	Glycinin G2 precursor	**EAFGVNMQIVR**	[1], [5], [18], [22]
	glycinin G4	HFLAQSFNTNEDIAEK	[1]
	glycinin G4 precursor	ISTLNSLTLPALR	[1], [16], [18],[20], [21], [24],

[1] Hoffmann, B.; Munch, S.; Schwagele, F.; Neususs, C.; Jira, W. A sensitive HPLC-MS/MS screening method for the simultaneous detection of lupine, pea, and soy proteins in meat products. *Food Control* **2017**, 71, 200–209, doi:10.1016/j.foodcont.2016.06.021.

[2] Huschek, G.; Bonick, J.; Lowenstein, Y.; Sievers, S.; Rawel, H. Quantification of allergenic plant traces in baked products by targeted proteomics using isotope marked peptides. *LWT-Food Sci. Technol.* **2016**, *74*, 286–293, doi:10.1016/j.lwt.2016.07.057.

[3] Chassaigne, H.; Norgaard, J.V.; van Hengel, A.J. Proteomics-based approach to detect and identify major allergens in processed peanuts by capillary LC-Q-TOF (MS/MS). *J. Agric. Food Chem.* **2007**, 55, 4461–4473, doi:10.1021/jf063630e.

[4] Heick, J.; Fischer, M.; Kerbach, S.; Tamm, U.; Popping, B. Application of a Liquid Chromatography Tandem Mass Spectrometry Method for the Simultaneous Detection of Seven Allergenic Foods in Flour and Bread and Comparison of the Method with Commercially Available ELISA Test Kits. *J. AOAC Int.* **2011**, 94, 1060–1068.

[5] Heick, J.; Fischer, M.; Popping, B. First screening method for the simultaneous detection of seven allergens by liquid chromatography mass spectrometry. *J. Chromatogr. A* **2011**, 1218, 938–943, doi:10.1016/j.chroma.2010.12.067.

[6] Pedreschi, R.; Norgaard, J.; Maquet, A. Current Challenges in Detecting Food Allergens by Shotgun and Targeted Proteomic Approaches: A Case Study on Traces of Peanut Allergens in Baked Cookies. *Nutrients* **2012**, 4, 132-150, doi:10.3390/nu4020132.

[7] Sayers, R.L.; Johnson, P.E.; Marsh, J.T.; Barran, P.; Brown, H.; Mills, E.N.C. The effect of thermal processing on the behaviour of peanut allergen peptide targets used in multiple reaction monitoring mass spectrometry experiments. *Analyst* **2016**, 141, 4130–4141, doi:10.1039/c6an00359a.

[8] Sayers, R.L.; Gethings, L.A.; Lee, V.; Balasundaram, A.; Johnson, P.E.; Marsh, J.A.; Wallace, A.; Brown, H.; Rogers, A.; Langridge, J.I., et al. Microfluidic Separation Coupled to Mass Spectrometry for Quantification of Peanut Allergens in a Complex Food Matrix. *J. Proteome Res.* **2018**, 17, 647–655, doi:10.1021/acs.jproteome.7b00714.

[9] Shefcheck, K.J.; Callahan, J.H.; Musser, S.M. Confirmation of peanut protein using peptide markers in dark chocolate using liquid chromatography-tandem mass spectrometry (LC-MS/MS). *J. Agric. Food Chem.* **2006**, 54, 7953–7959, doi:10.1021/jf060714e.

[19] New, L.S.; Schreiber, A.; Stahl-Zeng, J.; Liu, H.F. Simultaneous Analysis of Multiple Allergens in Food Products by LC-MS/MS. *J. AOAC Int.* **2018**, *101*, 132–145, doi:10.5740/jaoacint.17-0403.

[11] Zhang, J.S.; Hong, Y.W.; Cai, Z.X.; Huang, B.F.; Wang, J.L.; Ren, Y.P. Simultaneous determination of major peanut allergens Ara h1 and Ara h2 in baked foodstuffs based on their signature peptides using ultra-performance liquid chromatography coupled to tandem mass spectrometry. *Anal. Methods* **2019**, *11*, 1689–1696, doi:10.1039/c9ay00256a.

[12] Boo, C.C.; Parker, C.H.; Jackson, L.S. A targeted LC-MS/MS method for the simultaneous detection and quantitation of egg, milk, and peanut allergens in sugar cookies. *J. AOAC Int.* **2018**, *101*, 108–117, doi:10.5740/jaoacint.17-0400.

[13] Gu, S.Q.; Chen, N.N.; Zhou, Y.; Zhao, C.M.; Zhan, L.N.; Qu, L.; Cao, C.; Han, L.; Deng, X.J.; Ding, T., et al. A rapid solid-phase extraction combined with liquid chromatography-tandem mass spectrometry for simultaneous screening of multiple allergens in chocolates. *Food Control* **2018**, *84*, 89–96, doi:10.1016/j.foodcont.2017.07.033.

[14] Ogura, T.; Clifford, R.; Oppermann, U. Simultaneous detection of 13 allergens in thermally processed food using targeted LC-MS/MS approach. *J. AOAC Int.* **2019**, *102*, 1316–1329, doi:10.5740/jaoacint.19-0060.

[15] Pilolli, R.; De Angelis, E.; Monaci, L. In house validation of a high resolution mass spectrometry Orbitrap-based method for multiple allergen detection in a processed model food. *Anal. Bioanal. Chem.* **2018**, *410*, 5653–5662, doi:10.1007/s00216-018-0927-8.

[16] Pilolli, R.; De Angelis, E.; Monaci, L. Streamlining the analytical workflow for multiplex MS/MS allergen detection in processed foods. *Food Chem.* **2017**, *221*, 1747–1753, doi:10.1016/j.foodchem.2016.10.110.

[17] Korte, R.; Lepski, S.; Brockmeyer, J. Comprehensive peptide marker identification for the detection of multiple nut allergens using a non-targeted LC-HRMS multi-method. *Anal. Bioanal. Chem.* **2016**, *408*, 3059–3069, doi:10.1007/s00216-016-9384-4.

[18] Planque, M.; Arnould, T.; Dieu, M.; Delahaut, P.; Renard, P.; Gillard, N. Advances in ultra-high performance liquid chromatography coupled to tandem mass spectrometry for sensitive detection of several food allergens in complex and processed foodstuffs. *J. Chromatogr. A* **2016**, *1464*, 115–123, doi:10.1016/j.chroma.2016.08.033.

[19] Houston, N.L.; Lee, D.G.; Stevenson, S.E.; Ladics, G.S.; Bannon, G.A.; McClain, S.; Privalle, L.; Stagg, N.; Herouet-Guicheney, C.; MacIntosh, S.C., et al. Quantitation of Soybean Allergens Using Tandem Mass Spectrometry. *J. Proteome Res.* **2011**, *10*, 763–773, doi:10.1021/pr100913w.

[20] Planque, M.; Arnould, T.; Dieu, M.; Delahaut, P.; Renard, P.; Gillard, N. Liquid chromatography coupled to tandem mass spectrometry for detecting ten allergens in complex and incurred foodstuffs. *J. Chromatogr. A* **2017**, *1530*, 138–151, doi:10.1016/j.chroma.2017.11.039.

[21] Chen, S.M.; Yang, C.T.; Downs, M.L. Detection of Six Commercially Processed Soy Ingredients in an Incurred Food Matrix Using Parallel Reaction Monitoring. *J. Proteome Res.* **2019**, *18*, 995–1005, doi:10.1021/acs.jproteome.8b00689.

[22] Planque, M.; Arnould, T.; Delahaut, P.; Renard, P.; Dieu, M.; Gillard, N. Development of a strategy for the quantification of food allergens in several food products by mass spectrometry in a routine laboratory. *Food Chem.* **2019**, *274*, 35–45, doi:10.1016/j.foodchem.2018.08.095.

[23] Montowska, M.; Fornal, E. Absolute quantification of targeted meat and allergenic protein additive peptide markers in meat products. *Food Chem.* **2019**, *274*, 857–864, doi:10.1016/j.foodchem.2018.08.131.

[24] Montowska, M.; Fornal, E.; Piatek, M.; Krzywdzinska-Bartkowiak, M. Mass spectrometry detection of protein allergenic additives in emulsion-type pork sausages. *Food Control* **2019**, *104*, 122–131, doi:10.1016/j.foodcont.2019.04.022.

Supplementary Table S2. Marker peptides for alfalfa, broad bean, chickpea, lentil, lupine blue, lupine white, pea, peanut, and soy and their homologies (NCBI online search tool BLAST, parameters for database search: query cover = 100 %, percent identity = 100 %; without bacteria); *Target protein refer to *Medicago truncatula*.

Marker	Peptide sequence	Protein (NCBI)	Homologies (NCBI)
Alfalfa 1	VEGGLSIMSPPER	Legumin A (XP_024625506.1)*	*Trifolium pratense*
Alfalfa 2	FNLEAGDIMR	Sucrose-binding protein (XP_003590610.1)*	---
Alfalfa 3	ISDVNSLTLPILR	Legumin K (XP_003590684.1)*	---
Broad bean 1	EDVLSLAPK	Putative sucrose binding protein (CAC27161.1)	---
Broad bean 2	FNLEEGDLIR	Putative sucrose binding protein (CAC27161.1)	---
Broad bean 3	LSPGDVLVIPAGYPVAIK	Vicilin (1502201A)	---
Chickpea 1	GGLSFISPSEK	Legumin-like (XP_027188788.1)	---
Chickpea 2	IVDLAIPINTPAK	Provicilin-like (XP_004496703.1)	---
Chickpea 3	SSNPFTFLVPPR	Legumin-like (XP_027188788.1)	---
Lentil 1	VLLEDQEQEPQHR	Allergen Len c 1.0102 (Q84UI0)	---
Lentil 2	FFEVTPEK	Convicilin (CAP06309.1)	*Lens nigricans, Talaromyces stipitatus*
Lentil 3	VVDFVISLNRPGK	Convicilin (Q9M3X8)	
Lupine blue 1	QQEQQLEGELEK	Conglutin delta 3 (AEB33723.1)	---
Lupine blue 2	NTLEATFNTR	Conglutin beta (ACB05815.1)	*Lupinus albus*
Lupine blue 3	ISSVNSLTLPILR	Legumin J-like (XP_019429051.1)	---
Lupine white 1	NPYHFSSQR	Conglutin beta 5 (KAE9612200.1)	---
Lupine white 2	DKPSQSGPFNLR	Conglutin beta 1 (KAE9612202.1)	---
Lupine white 3	AVNELTFPGSAEDIER	Conglutin beta 2 (Q6EBC1.1)	---
Pea 1	ELTFPGSVQEINR	Convicilin (CAB82855.1)	---
Pea 2	LSSGDVFVIPAGHPVAVK	Vicilin (P13918.2)	---
Pea 3	LTPGDVFVIPAGHPVAVR	Provicilin (P02855.1)	---
Peanut 1	GTGNLELVAVR	Allergen Ara h 1 (ADQ53858.1)	*Arachis ipaensis, Arachis duranensis*
Peanut 2	FNLAGNHEQEFLR	Allergen Ara h 3/ h 4 (AAM46958.1)	*Arachis ipaensis, Arachis duranensis*
Peanut 3	WLGLSAEYGNLYR	Allergen Ara h 3/ h 4 (AAM46958.1)	*Arachis ipaensis, Arachis duranensis*
Soy 1	SQSDNFEYVSFK	Glycinin G1 precursor (NP_001235827.2)	*Glycine soja*
Soy 2	EAFGVNMQIVR	Glycinin G2 precursor (NP_001235810)	*Glycine soja*
Soy 3	FYLAGNQEQEFLK	Glycinin G1 precursor (NP_001235827.2)	*Glycine soja*

Supplementary Table S3. Groups of possible ingredients for the production of sausages and commercial spice mixtures, which were tested regarding cross-reactivity with the legume marker peptides analyzed

**Group 1**	**Group 2**
cauliflower (*Brassica oleracea var. botrytis*)	leek (*Allium ampeloprasum*)
broccoli (*Brassica oleracea var. italica Plenck*)	onion (*Allium cepa*)
brussel sprouts (*Brassica oleracea var. gemmifera*)	rape (*Brassica napus*)
kohlrabi (*Brassica oleracea var. gongylodes*)	spring onion (*Allium fistulosum*)
savoy cabbage (*Brassica oleracea convar. capitata var. sabauda*)	swede (*Brassica napus subsp. rapifera*)
	wild garlic (*Allium ursinum*)

**Group 3**	**Group 4**
almond (*Prunus dulcis*)	beetroot (*Beta vulgaris*)
brazil nut (*Bertholletia excelsa*)	carrot (*Daucus carota*)
cashew (*Anacardium occidentale*)	celery (*Apium graveolens*)
hazelnut (*Corylus avellana*)	horseradish (*Armoracia rusticana*)
macadamia (*Macadamia spp.*)	radish (*Raphanus spp.*)
pistachio (*Pistacia vera*)	

**Group 5**	**Group 6**
cucumber (*Cucumis sativus*)	asparagus (*Asparagus officinalis*)
pumpkin (*Curcubita spp.*)	fennel (*Foeniculum vulgare*)
tomato (*Lycopersicon esculentum*)	ginger (*Zingiber officinale*)
zucchini (*Curcubita pepo*)	marjoram (*Origanum majorana*)
paprika (*Capsicum annuum*)	parsley (*Petroselinum crispum*)
champignon (*Agaricus spp.*)	
chili (*Capsicum spp.*)	

**Group 7 (curry spice ingredients)**	**Commercial spice mixtures for the use in…**
black pepper (*Piper nigrum*)	Bavarian veal sausages
caraway (*Carum carvi*)	"Bratwurst"
cardamom (*Elettaria cardamomum*)	Ham sausages
clove (*Dianthus*)	Curry (for various sausages)
coriander (*Coriandrum sativum*)	Emulsion-type sausages ("Lyoner")
fenugreek (*Trigonella foenum-graecum*)	Frankfurter-type sausages
garlic (*Allium sativum*)	"Gelbwurst"
lovage (*Levisticum officinale*)	Krakauer/Bierwurst
mace (*Myristicae arillus*)	Liver sausage
nutmeg (*Myristica fragrans*)	"Pressack" white
turmeric (*Curcuma longa*)	Blood sausages/ "Pressack" red
white mustard (*Sinabis alba*)	

Supplementary Table S4. Normalized peak areas of legume marker peptides expressed as a percentage of the most intense marker for a species (= 100 %; marked in bold), CVs of the peak areas, means and standard deviation mass transitions, and CVs of the ratio of peak area of the lowest to the highest intense mass transition (N = 70, each).

Marker Peptide	Normalized peak area (%)	CVs peak area (%)	Means and standard deviation mass transition ratio	CVs mass transition ratio (%)
**Alfalfa 1**	100	7	0.426 ± 0.017	4
Alfalfa 2	9	29	0.520 ± 0.104	20
Alfalfa 3	9	27	0.478 ± 0.043	9
Broad bean 1	33	11	0.422 ± 0.021	5
Broad bean 2	19	12	0.664 ± 0.056	8
**Broad bean 3**	100	12	0.115 ± 0.003	3
Chickpea 1	33	9	0.706 ± 0.025	4
Chickpea 2	14	16	0.456 ± 0.019	4
**Chickpea 3**	100	17	0.067 ± 0.004	5
Lentil 1	42	22	0.229 ± 0.028	12
**Lentil 2**	100	16	0.265 ± 0.022	8
Lentil 3	62	28	0.132 ± 0.015	12
**Lupine blue 1**	100	8	0.301 ± 0.007	2
Lupine blue 2	72	23	0.830 ± 0.087	10
Lupine blue 3	12	34	0.110 ± 0.027	24
Lupine white 1	84	11	0.234 ± 0.014	6
**Lupine white 2**	100	19	0.391 ± 0.009	2
Lupine white 3	4	80	0.442 ± 0.049	11
Pea 1	17	12	0.287 ± 0.016	5
Pea 2	14	11	0.720 ± 0.032	6
**Pea 3**	100	12	0.198 ± 0.006	3
Peanut 1	18	15	0.640 ± 0.042	7
Peanut 2	92	8	0.257 ± 0.011	4
**Peanut 3**	100	24	0.451 ± 0.011	2
Soy 1	17	18	0.267 ± 0.017	6
Soy 2	50	11	0.651 ± 0.024	4
**Soy 3**	100	9	0.151 ± 0.006	4
